# Considerations for prioritising clinical research using bacteriophage

**DOI:** 10.1042/EBC20240013

**Published:** 2024-12-17

**Authors:** Sarah J.L. Edwards, Yiran Tao, Rodas Elias, Robert Schooley

**Affiliations:** 1 STEaPP, University College London, 255309, Gower Street, London, U.K.; 2 STS, University College London, 265201, London, U.K.; 3 STS, University College London, 265202, London, U.K.; 4 Division of Infectious Diseases, University of California San Diego, 265203, San Diego, California, U.S.A.

**Keywords:** bacteriophages, Clinical trials, funding priorities, regulation

## Abstract

Antimicrobial resistance (AMR) poses a significant global health threat, as it contributes to prolonged illness, higher mortality rates and increased healthcare costs. As traditional antibiotics become less effective, treatments such as bacteriophage therapy offer potential solutions. The question remains, however, on how to set research priorities in the face of a growing number of antibiotic-resistant pathogens, some common and/or dangerous. One standard way of making decisions about which research to prioritise is by using the disability-adjusted life year metric to estimate the current global impact of a disease or condition, combined with considerations of social justice although decisions made at a national level by governments, especially in low income countries with forecasting potential over future needs may look very different. Another approach is based on the needs of researchers and regulators given what we know about the technology itself. The biological characteristics of bacteriophage therapies set challenges to a universal and standardised prioritisation method. A proof of principle is still arguably needed. With a preliminary discussion of the scope and complexity of AMR and AMR therapeutics, we propose some implications of regulatory frameworks aiming to integrate bacteriophage therapy into mainstream medical practice while gathering scientific data on safety and efficacy, enhancing the collective action needed to combat AMR.

## Introduction

The growing threat of antimicrobial resistance (AMR) is one of the major global health challenges of our time. Such resistance can lead to prolonged illnesses, increased healthcare costs, and higher mortality rates. Annually, AMR is already directly responsible for approximately 1.2 million deaths around the world and is associated with approximately 5 million deaths [1]. While precise projections vary, there is a near consensus over slowly progressing yet impending doomsday scenarios. Some estimates suggest that without reversing this trend, AMR could lead to 10 million deaths a year by 2050, significant disruption to common surgical and medical interventions, and a further 24 million people driven into extreme poverty. It is estimated to lead to a global annual GDP loss of between 1.1% and 3.8% by 2050, with an annual shortfall of up to USD 3.4 trillion by 2030 [2]. Addressing AMR is thus crucial to reducing the burden of disease and improving public health which requires a comprehensive approach that includes promoting appropriate antibiotic use, implementing infection prevention and control measures, developing new antimicrobial drugs, and investing in research and development of alternative treatments, such as bacteriophage therapy, to combat drug-resistant infections. The call for public health practitioners, policymakers, and research organisations to prioritise innovation in science and technology is therefore becoming more urgent. Indeed, the World Health Organization (WHO) published a policy brief in 2023 to outline global research priority topics for tackling AMR in human health with a timeline to inform policy by 2030 [3]. In particular, the policy brief outlines forty priority topics including the investigation of safe and efficacious antibiotic treatment regimens based on both new and old agents in various combinations for infections, especially for extended-spectrum beta-lactamase-producing and/or carbapenem-resistant Enterobacterales, with minimal selection and transmission risk for developing further AMR, especially among children and other subpopulations experiencing vulnerability [3]. The brief continues to identify investigations of safe and efficacious antibiotic treatments (drug choice, drug combination, route, dose and duration) for gram-negative bacteria causing bloodstream infections or sepsis among neonates and young children, especially in settings with high prevalence of AMR, restricted diagnostic capacity, and low availability of antimicrobial medicine. Both priority areas are underpinned by legal rights to health which gives children special protective status and confers obligations on governments to pursue research and development and collaborate across jurisdictions [4]. While the pandemic has raised both more respect for and mistrust in science and technology, such policies provide a framework within which to encourage, even incentivise collective action [5].

This paper seeks to focus attention specifically on the prospects for bacteriophages in these endeavours by exploring how we might think about setting priorities for bacteriophage research to contribute to what has to be collective action. Bacteriophages are viruses which kill bacteria and have been routinely prescribed in some countries which did not adopt antibiotics as did countries in the West, as they were too expensive and were deemed unnecessary [6]. However, the commercial models behind developing new medicines, epistemic standards of evidence-based medicine, and regulatory rules to market medicines have largely prevented them being adopted in the West [7]. That said, there is renewed interest in using bacteriophage as therapy with some clinical trials already recruiting patients while early or expanded access programmes support ‘compassionate use’ in the absence of clinical trials either using standardised protocols for aggregate monitoring and analysis using real-world data or as ad hoc therapies for individual patients [8]. We think a conversation about priorities for clinical research of bacteriophage is now urgent. Such a conversation should take into account the nature of the technology, as well as which standards of evidence in medicine and associated regulatory pathways are most suitable.

## Current clinical research landscape

Over recent years there has been a marked increase in the number of clinical trials using bacteriophage either in combination with antibotics or as single interventions [9]. As of July 2024, there were nearly 100 studies listed on clinicaltrials.gov from searching for ‘bacteriophage’ or ‘phage therapy’ as ‘other terms’, of which about two-thirds were listed as interventional, the rest being observational or expanded access programmes. Roughly half were funded by industry. Nearly a third had been completed, while roughly a fifth had been withdrawn, were no longer recruiting or had been terminated. Roughly a third were actively recruiting openly or by invitation, or were about to start recruiting. Two-thirds are listed as phase 1 and 2, with a handful as phase 3 and 4, while many were not catergorised by phase at all. Only one of the later phase trials was a placebo-controlled trial with modest expected sample size of 50 patients with urinary and vaginal infections. The most active areas of research registered were for use against infections of prosthetic joints, the urinary tract, and with underlying cystic fibrosis.

Despite growing interest, reviews of available published clinical trial results have largely reported safety data only, with mixed or inconclusive results for efficacy where reported [8,10]. A published review of results for priority pathogens causing multidrug resistant infections included 30 studies [11]. Despite covering more than 1152 patients overall, many were case studies or observational cohorts of patients infected with antibiotic resistant *E. coli*, with *S. aureus*, with *K. pneumoniae*, with *A. baumannii*, and with *P. aeruginosa*. There are now increasing numbers of studies addressing priority pathogens, including one active clinical trial in Phase 3, albeit single armed, to treat antibiotic resistant tonsillitis in children [12,13]. A cohort study over two time periods and two early phase clinical trials address antibiotic-resistant *S. Aureus* infections [14–17]. In six of the early phase eight cystic fibrosis trials, bacteriophage therapy targets *P. aeruginosa*, a critical priority pathogen for AMR and a leading cause of complications and death [18]. While four trials have been completed, two have not yet published results, and the remaining two, with fewer than five participants each, yielded inconclusive findings. Two larger scale (<50 participant) studies are actively recruiting to carry out studies into the effectiveness of bacteriophage therapies in cystic fibrosis.

## Prioritisation methods

There is significant heterogeneity in approaches to setting health research priorities [19–21]. In addition to variable methods, there is a lack of standardised reporting. Furthermore, there is no agreement on which is the best method or group of methods [22,23]. The most suitable methods are likely to be those which suit the specific purposes of the priority setting activities, are appropriate for the research context or ecosystem, and take account of the available resources. However, the context in which priorities are set can themselves be changed if deemed unsuitable. We will first outline the current context before identifying features of bacteriophage technology and the context of the AMR threat which might prompt us to recommend certain changes to our methodological approach to clinical research and the regulation within which such research can be supported.

Clinical research is conventionally seen as most socially valuable in areas where current treatment options are poor or ineffective. It thus seems reasonable to focus on diseases or conditions where bacteriophage therapy has the potential to provide the most significant clinical benefits. The traditional approach to prioritising research has been to minimise global burden of disease using the DALY metric (disability-adjusted life year) to achieve a combined assessment of morbidity and mortality [23]. This metric is used to estimate the impact of a disease or condition on a population by combining the number of years of life lost due to premature death and the number of years lived with disability or poor health. DALYs provide a way to quantify and compare the burden of different diseases and conditions on a population.

AMR certainly has a significant impact on DALYs, as it can lead to prolonged illness, treatment failures, and increased mortality rates. The rise in AMR has already been identified as a global public health threat, as it can undermine the effectiveness of many other medical or surgical interventions, including the treatment of infectious diseases. As a result, AMR contributes to an increase in the overall burden of disease, as measured by DALYs, yet early efforts to quantify the precise impact of AMR on DALYs have been restricted to certain European jurisdictions. Better reporting in surveillance studies especially in low income countries is needed to better model the exact global impact AMR has now and can be expected to have in the coming years [24,25]. However, while DALY scores are based on consensus discussions amongst patients, clinicians and researchers, they are recognised as potentially reinforcing unfair allocation of resources in research by including assumptions such as time-discounting and age weighting [26]. Attempts to remove such evaluative assumptions leading to these results though have been only partially successful in making them more descriptive [26]. Built on consequentialist methods, the action which reduces the most DALYS, however they are distributed across the population is, by definition, regarded as the morally right course to take. Those with rare diseases may thereby be disadvantaged unless grouped together as a class to compete with more common diseases unless the rare disease in question sufficiently alters the average score [27]. More recently and in recognition of such difficulties, WHO published a guide for its staff to help make prioritising clinical research more systematic and now explicitly includes considerations of equity and universal healthcare coverage which go beyond using a simple DALY model [23].

Separately, WHO has identified priority pathogens to guide and promote research and development (R&D) of new antibiotics as part of WHO's efforts to address growing global resistance to antimicrobial medicines [13]. The list is divided into three categories according to the perceived urgency of need for new antibiotics, i.e., critical, high, and medium. The most critical group of all includes multidrug resistant bacteria that pose a particular threat in hospitals, nursing homes, and among patients whose care requires devices such as ventilators and blood catheters. They include *Acinetobacter*, *Pseudomonas* and various Enterobacteriaceae (including *Klebsiella*, *E. coli*, *Serratia*, and* Proteus*). They can cause severe and often deadly infections of the lungs and bloodstream infections. These bacteria have become resistant to a large number of antibiotics, including carbapenems and third generation cephalosporins - previously the best available antibiotics for treating multidrug resistant bacteria. The second and third tiers in the list - the high and medium priority categories - contain other increasingly drug-resistant bacteria that cause more common diseases such as gonorrhoea and food poisoning caused by *salmonella* Table 1.

**Table 1:** WHO list of priority pathogens for R&D 2017

**Priority 1: CRITICAL**
● *Acinetobacter baumannii*, carbapenem-resistant
● *Pseudomonas aeruginosa*, carbapenem-resistant
● *Enterobacteriaceae*, carbapenem-resistant, ESBL-producing
**Priority 2: HIGH**
● *Enterococcus faecium*, vancomycin-resistant
● *Staphylococcus aureus*, methicillin-resistant, vancomycin-intermediate and resistant
● *Helicobacter pylori*, clarithromycin-resistant
● *Campylobacter spp.*, fluoroquinolone-resistant
● *Salmonellae*, fluoroquinolone-resistant
● *Neisseria gonorrhoeae*, cephalosporin-resistant, fluoroquinolone-resistant
**Priority 3: MEDIUM**
● *Streptococcus pneumoniae*, penicillin-non-susceptible
● *Haemophilus influenzae*, ampicillin-resistant
● *Shigella spp.*, fluoroquinolone-resistant

A recent review by the International Federation of Pharmaceutical Manufacturers and Association (IFPMA) of the pipeline for new antibiotics against bacterial pathogens identified by WHO as of the greatest concern and a further three pathogens identified by other public health agencies show that it is not nearly sufficient [27]. There have been only ten new antibiotics or combinations approved by regulatory authorities between 2017 and 2023, only two of which are defined as truly innovative by the WHO. None are considered to constitute a new class of antibiotics. There is currently just one antibiotic candidate in phase 3 clinical trials across the four bacterial pathogens defined as a critical priority by WHO. Just two of the seven high-priority pathogens have innovative candidate antibiotics in development, with five having three or fewer candidates at any stage of clinical development.

‘Pull’ incentives are now widely considered necessary to improve the above pipeline [28]. The total amount needed to provide a sufficient return on successful development and thereby incentivise private investment has been well-studied and quantified [29]. By unlocking additional investment from private investors, supported by continued public/private ‘push’ funding, the recent analysis in the above review shows a possible nineteen new approvals by 2033, compared with only eight otherwise. Beyond approved treatments, the IFPMA further shows that such incentives could have a significant impact on the number of candidates in clinical trials. In 2033, the pipeline could consist of 72 treatments, of which 41 could be in the late stages.

To estimate the potential population health benefits from a more robust pipeline of antibiotics, the recent report also separately modelled the expected DALY impact of four WHO critical priority pathogens namely *Acinetobacter baumannii, Escherichia coli, Klebsiella pneumoniae, Pseudomonas aeruginosa*, using two different assumptions. Without new incentives and with no new antibiotics to treat these resistant infections, the burden in high-income countries (HICs) where surveillance data are greatest would be expected to increase by about 35% on average in 10 years compared to today. If these incentives were to be introduced, and new antibiotics against these pathogens approved by regulators as a result, these could be expected to reduce the DALY score by more than 50%. While this impact was only modelled for HICs due to limitations in surveillance data, a similar benefit might be expected to translate to low-income settings [28].

Futhermore, funding decisions for research made by governments will often focus on modelling national rather than international prevalence of diseases so distort the application of DALYs even with adjustments for social justice. For example, many of the priority pathogens are particular problems in low income settings and not apparently an immediate threat to more developed countries. However, along with discussions over drawing up a pandemic treaty and the extent to which global travel may or may not affect the transmission of AMR genes, the international community through the UN and WHO offer some ability to coordinate research activities and assess national ‘preparedness’ for future public health crises of one sort or another.

Ultimately, however, when prioritising research in science and technology, there are more considerations than simply responding to the highest clinical need, such as those diseases associated with high mortality rates and for which there is no existing effective treatment. Given the current status of the technology of bacteriophage in different countries, its unique features making them inevitably more numerous and personalised in nature. There is little sign of developing a comprehensive and coordinated roadmap for research. Despite widespread use in some countries for common, if mild to moderate infections, there still lacks the clinical evidence regulators and payers would want to see before adopting similar regimens wholesale in Western jurisdictions. Remarkably, there is still a perceived need for a proof of principle given such mixed clinical data as described above to support their wider development and evaluation before offering for routine use.

As there is considerable anecdotal and observational data to support the safety and use of certain phages for common infections, we could set out simply to bolster the evidence base and head straight for pivotal confirmation trials of existing regimens used routinely elsewhere. This may help ensure that the therapy is more likely to be successful in clinical trials. We might similarly prioritize clinical research on bacteriophage therapy that aligns most closely with current regulatory requirements. This should help facilitate the approval and adoption of bacteriophage therapy as a viable treatment option. Considered under the classification of a biological medicine, there are general regulations which apply if no specific guidance for bacteriophage. Clinical trial paradigms have been well worked out for traditional antibiotics so following those paradigms as closely as possible would arguably make it easier for regulators and others to understand the impact of bacteriophages in the context of the results antibiotics deliver to similar clinical entities.

## Particular features of bacteriophage make standard approaches to R&D for pharmaceuticals difficult

The number of different therapeutically interesting phages is too vast to be able to fully test each individually and separately even once they have been discovered and purified to any agreed standard [30]. We are left trying to optimise a proposed intervention in the laboratory through in vitro experiments and use of AI to model and screen candidate bacteriophages trained on existing data such as they are. There is thus far more work on the underlying biology required than a rather hit and miss approach sometimes underpinning clinical trials. For example, some uses of whole bacteriophages or their enzymes may be designed to work alongside standard antibiotics as a combination therapy to help disarm the pathogen first ready for the once failing antibiotics to regain the upper hand [30].

Another biological feature that poses challenges is the narrow host range of each bacteriophage, meaning that it can target only a small number of bacterial species or, indeed, strains. Moreover, bacteriophages and their hosts evolve at a high speed as they interact, which may result in phage-resistant pathogens. Thus, different bacteriophages may be mixed into a phage cocktail and tested for possible resistance before used in clinical settings. Phage cocktails that cover most strains have been tested only for a few albeit common bacterial pathogens. However, the PhagoBurn study on *Pseudomonas* was ultimately unsuccessful. It is possible to develop standardised phage cocktail treatment for some pathogens, allowing for a straightforward trial in which a fixed phage regimen is treated the same way one would a novel antibiotic. Bacteriophage therapies for other pathogens remains individualistic until the determinants of their phage host range are discovered. Considering how host range impacts the therapeutic potential of bacteriophages, scientific and engineering efforts should be guided according to the current understanding of this aspect of phage and preclinical studies to identify and overcome potential emerging resistance to phage regimens [30].

However, there are also studies showing that straightforward translation from laboratory results to clinical results are similarly challenging which reinforces the case for bolstering the clinical evidence needed before widespread adoption. At present, clinical trials have thus far showed mixed results and implementing clinical trial design that supports the optimum efficacy of bacteriophages is needed [30,31]. This includes the use of phages prepared against an agreed standard with central reference phages to ensure phage quality and consistency in trials as well as using personalised phage cocktails [32]. There are thus open questions over the best design to demonstrate safety and efficacy as well as the most suitable regulatory pathway to include early or expanded access. However, a major hurdle is still the upfront investment needed before such a proof of principle can be established. Even at the smallest scale, there are challenges in meeting regulatory standards of production such as good manufacturing practice [33].

## Ways forward?

Traditional research and development especially in industrial settings has sought to identify the single best candidate to take forward in a series of clinical trials as required for a market license [34]. Such a strategy puts method and scientific norms in medicine above context and the nature of the technology itself. While the COVID-19 pandemic has challenged many of these traditions, there are still regulatory and commercial barriers to designing studies which best suit particular technologies [35] Generally speaking, it would seem impossible to use the traditional strategy of identifying a single best bacteriophage regimen to be able to test in a traditional placebo controlled RCT, if only because a biological medicine hinges on host response which is inevitably heterogenous. That said, there are many more tools available for research and development, such as high-throughput methods for efficiently screening thousands of samples at the same time, affordable whole-genome sequencing, automated technology for microbiological techniques. The application of artificial intelligence could also facilitate the selection of phages depending on the specific characteristics of the target pathogens and the host's profile [30].

Established advanced cellular therapies are similarly personalised presenting challenges for regulators in appraising evidence despite the considerable commercial interest in these technologies [36]. Many pivotal phase 3 trials of such therapies have not been randomised controlled trials but have been informed by genetic profiling of the host to fit particular cases [35,37,38]. Here the clinical investigation is tailored to the strength of the preclinical data and mechanistic understanding. Early or expanded access programmes have sometimes required stronger surveillance later to compensate for weaker evidence of clincial outcomes from clincial trials [39]. It is less clear though whether such an approach could be taken with bacteriophage given that there is currently less compelling mechanistic evidence to support such an epistemic and regulatory shortcut in clinical evidence.

Sometimes, the need for clinical research is secondary to the therapeutic rationale. For example, Phage Australia have set up such a registry with substantial government funding to collect and rely solely on observational data for all the off-label uses of bacteriophages they support for aggregate monitoring and real-world analysis [40]. The use of real-world evidence for regulatory decision-making in general is both promising and contested with most regulators still prioritising clinical trials [41]. Similarly, a retrospective analysis of a cohort of patients with infections which were difficult to treat with antibotics were treated with bacteriophage through a consortium of hospitals has recently been published [42]. However, observational data alone may not be persuasive. Rather than to evaluate each intervention separately at large scale to provide evidence for demonstrating the single best, there are more diverse methods already being used to evaluate an array of possible interventions for AMR infections. For pathogens in which the host range is more complicated, the set of conditions that might predict success for a personalized phage cocktail could first be identified (e.g., two or more phages with lytic activity [defined in a well described and standardized *in vitro* assay] against the pathogen). Bacteriophage combinations satisfying those criteria could technically be treated as a ‘phage therapy arm’ that could be used in a traditional placebo controlled or placebo plus standard of care vs phage trial. However, as in other examples where a disease is life-threatening, there is much disagreement over the ethics of restricting new treatments to randomized controlled trials giving a proportion of patients treatment known to be ineffective. Alternatively, the personalised clinical trial approach seeks simply to record whether or not there is an effect and ultimately to rank numerous available interventions from the range clinically feasible at the time of treatment rather than to estimate precise effect sizes for each [43]. This design, called the PRACTical trial design, offers a new approach inspired by network meta-analysis which compares multiple treatments in an evidence synthesis, to identify, what is the qualitatively the best treatment out of a set of available treatments. Statistical methods have advanced to be able to make indirect comparisons to compare relative effects of different regimens across different clinical trials. When used to design a clinical trial, the principle potentially helps balance the rights and interests of individual patients along with clinician's judgement of plausibility and therapeutic preference, with scientific validity through randomized controlled trials given numerous treatment possibilities under uncertainty. This design thus does not rise the same ethical concerns with clinicians suspending all their initial treatment preferences for the sake of holding clinical equipoise, a state of epistemic uncertainty amongst the community of clinicians, which helps justify randomisation. Choosing a regimen that is likely to be one of the best of the available options at that time for the individual patient is thus more important than finding the perfectly optimal regimen overall. These are trade-offs clinicians may want to make to personalise decisions for each individual patient in balancing factors such as efficacy, toxicity, resistance, availability and cost. Bacteriophage combinations satisfying those above criteria would be entered as any number of additional intreventions in the PRACTical trial design. A similar design may be used to randomize patients to routinely available treatments where the relative effects of these treatments at a population level are unknown. Theoretical equipoise where the individual clinician is uncertain of relative treatment effects of a sub-set of therapeutic options provides boundaries for deciding which interventions to allocate randomly to a particular patient. A combination of methods would seem to point to making the most robust inferences about cause and effect [44], yet we need to establish a clear and coordinated way forward to give the technology the best chance to confer clinical benefits and to determine its prospects for combatting AMR.

## Conclusion

We call for more concerted attention on setting priorities for clinical research to take account of the unique features of the technology and the context within which it exists. The phage community, clinicians and regulators should now reach beyond debates around regulatory standards for manufacture, for early or expanded access to bacteriophage and the need for more evidence. We need to agree an approach which will reconcile competing pressures at the same time as recognise the unique feature of the technology and the integral role of basic science in guiding the way for public health.

## Summary

- AMR is responsible for approximately 1.2 million deaths annually and could cause up to 10 million deaths per year by 2050 if the trend continues.
- Bacteriophages, viruses that prey on bacteria, present a promising addition to antibiotics, especially for drug-resistant pathogens.
- The traditional prioritisation method using the DALY metric has yet to be successfully applied to AMR on a global scale, calling for a more nuanced approach that includes social justice, equity and universal healthcare coverage.
- Unique features of bacteriophages, such as their narrow host range and rapid evolution, complicate standard R&D methodologies yet suitable methods are available.
- A coordinated effort among researchers, clinicians, and regulators however is required to overcome barriers to developing novel therapeutics such as bacteriophages. Specifically, regulatory frameworks that meet the needs of bacteriophages are essential.

## Open Access

Open access for this article was enabled by the participation of University College London in an all-inclusive *Read & Publish* agreement with Portland Press and the Biochemical Society under a transformative agreement with JISC.

## Abbreviations

AMR: antimicrobial resistance
DALY: disability-adjusted life year
HIC: high-income country
